# Household medical waste disposal policy in Israel

**DOI:** 10.1186/s13584-016-0108-1

**Published:** 2016-10-10

**Authors:** Zohar Barnett-Itzhaki, Tamar Berman, Itamar Grotto, Eyal Schwartzberg

**Affiliations:** 1 Public Health Services, Ministry of Health, 39 Yirmiyahu St, Jerusalem, Israel; 2 Mimshak, The Israel society of ecology and environmental sciences, 19 Kehilat New York St, Tel Aviv, Israel; 3 Faculty of Health Sciences, Ben-Gurion University of the Negev, P.O.B. 653, Beer-Sheva, Israel; 4 Pharmaceutical Division, Ministry of Health, 39 Yirmiyahu St, Jerusalem, Israel

**Keywords:** Pharmaceutical waste, Environment, Return Unwanted Medicines, Medication, Pollution, Polluter pays

## Abstract

**Background:**

Large amounts of expired and unused medications accumulate in households. This potentially exposes the public to hazards due to uncontrolled use of medications. Most of the expired or unused medications that accumulate in households (household medical waste) is thrown to the garbage or flushed down to the sewage, potentially contaminating waste-water, water resources and even drinking water. There is evidence that pharmaceutical active ingredients reach the environment, including food, however the risk to public health from low level exposure to pharmaceuticals in the environment is currently unknown. In Israel, there is no legislation regarding household medical waste collection and disposal. Furthermore, only less than 14 % of Israelis return unused medications to Health Maintenance Organization (HMO) pharmacies.

**Methods:**

In this study, we investigated world-wide approaches and programs for household medical waste collection and disposal.

**Results:**

In many countries around the world there are programs for household medical waste collection. In many countries there is legislation to address the issue of household medical waste, and this waste is collected in hospitals, clinics, law enforcement agencies and pharmacies. Furthermore, in many countries, medication producers and pharmacies pay for the collection and destruction of household medical waste, following the “polluter pays” principle.

**Conclusions:**

Several approaches and methods should be considered in Israel: (a) legislation and regulation to enable a variety of institutes to collect household medical waste (b) implementing the “polluter pays” principle and enforcing medical products manufactures to pay for the collection and destruction of household medical waste. (c) Raising awareness of patients, pharmacists, and other medical health providers regarding the health and environmental risks in accumulation of drugs and throwing them to the garbage, sink or toilet. (d) Adding specific instructions regarding disposal of the drug, in the medication label and leaflet. (e) Examining incentives for returning medications to pharmacies. (f) Examining drug collection from deceased in retirement homes and hospitals.

**Electronic supplementary material:**

The online version of this article (doi:10.1186/s13584-016-0108-1) contains supplementary material, which is available to authorized users.

## Introduction

Significant developments in the drug industry, in addition to the improved access of a variety of populations to western medications have contributed to the increase in medication consumption all over the world [1, 2]. This trend is expected to extend in the next years, due to the growth in the population and in the access of additional populations to medications, in particular western medications.

Currently, large amounts of expired and unused medications accumulate in households, both prescription and over-the-counter (OTC) medications. Most of these household expired or unused medications (household medical waste) are thrown to the garbage or flushed down the sink or the toilets. Polls conducted in 2009 in Israel and in 2010 in Europe show that 50 % of the European public and 84 % of the Israeli public throw household medical waste to the garbage or flush them down the toilet [3, 4]. In Israel, results of a survey in a nationally representative sample (*N =* 602) showed that 80 % of responders throw medications to the garbage; 4 % flush them down toilets; and only 6 % return them to the doctor or pharmacy or donate them to the needy [4].

Accumulation of medications in private homes exposes the public to medical hazards: accumulation of medications increases the chance of uncontrolled use of the medication and subsequent poisoning.

More than 30 % of the poison cases among children in the U.S on 2009 were caused by uncontrolled use of medications [5]. According to the Israel poison information center at Rambam hospital, 43 % of the applications refer to children under six years old, and about half of the applications refer to exposure to medications [6]. Medication reserves can also be used for abuse or suicide.

Throwing household medical waste to the garbage or to the sewage can also cause environmental hazards that may have implications on the public health: some of the medications consist of stable ingredients that may accumulate in the environment and are barely removed in wastewater treatment plants [7]. Therefore, residues of medications may contaminate purified wastewater that are used for agricultural irrigation (more than 90 % of the wastewater in Israel is treated in treatment plants, most of which is used for agriculture). In recent years, researchers have found that agricultural products irrigated with treated wastewater contain pharmaceutical compounds. Of note, some of the medications found in wastewater are excreted from humans and animals and not from thrown medications.

The pharmaceutical compounds may contaminate water reservoirs and aquifers, and pollute drinking water, in addition to contamination of ecological systems [8]. Data shows that pharmaceuticals are present in drinking water sources [9].

Furthermore, throwing antibiotics to the sewage may lead to selective pressure and contribute to development of resistance to antibiotics among populations of bacteria [10]. In 2012, antibiotic resistance genes were found in bacteria taken from six rivers in China, including the Pearl River which is considered the river with the highest pollution of antibiotics in China [11]. The rise of antibiotic resistant bacteria and genes, is a major problem in today’s medicine and may significantly increase the number of deaths due to infectious diseases [12].

Here we investigated programs and actions for household medical waste collection and disposal around the world, in order to suggest approaches to improve the situation in Israel.

## Methods

We searched PubMed and Google for reviews, papers, reports and articles, published between January 2009 and May 2016 in English, related to medicines and drugs (terms “medical” or “medication” or “pharmaceuticals” or “drug”), and household (“term household”), and disposal (terms “waste” or “disposal” or “disposed”). We focused on the household medical waste policies in Europe, Canada, USA, South and Central American representative countries, Australia, Middle East representative countries (Lebanon, Egypt and Saudi Arabia), and Israel. In addition, we searched for relevant information in the following websites: World Health Organization, US Environmental Protection Agency, US Drug Enforcement Agency, US Food and Drug administration, Health Canada, the Israeli Ministry of Health, and Israeli HMOs. We also used information from additional health and environmental organization websites, and from international pharmaceutical collection program websites.

### World-wide approaches and programs for drug collection and disposal

#### Europe

##### Policy

An European Union (EU) directive from 2001 (DIRECTIVE 2001/83/EC) stated that special precautions should be used for disposal of unused medicinal products [13]. It was stated that the special precautions for disposal must appear on the outer packaging of medicinal product [13]. In 2004, two additional EU directives stated that: (a) EU states should establish medication collection systems, (b) information regarding the collection system in the specific state or county must appear on the outer packaging of the medicinal product [14].

Today, most of the European countries supply detailed information regarding the collection of unused/expired medications. Information channels range from oral information to the patient by the physician or the pharmacist on the best way of disposal, brochures, comprehensive information via websites, information on collection containers and information on the package of the pharmaceutical product [3]. Of note, besides Malta, Slovenia and North Rhine Westphalia state of Germany, all states and regions classify pharmaceutical waste as special waste that should be returned to a pharmacy [3].

##### Medication collection systems

Different states in Europe have different policies regarding household medical waste collection:

- Several states in Europe have legislation obliging pharmacies to collect household medical waste: Iceland, Estonia, Belgium, UK, Denmark, Lithuania, Lichtenstein, Norway, France, Hungary, and Croatia [3, 15] (see Table 1). In the UK, the National Health Service (NHS) is in charge of collection of medical waste from pharmacies that were returned by households and residential homes [16]. In France, collected drugs that were still usable were used to be redistributed to humanitarian associations [17].

**Table 1:** Comparison between the policies, collection and funding systems, regarding household medical waste in six selected countries

Country	National drug collection program	Legislation	Collection method	Funding	Medical collected (weight)/collection rates (annual)
USA	Some programs are run by the DEA [23], specific counties [25], and by local communities [42]	The law enables hospitals, clinics, pharmacies and drug producers to collect unused drugs [22]. In several counties and in one state (MA) there are legislations regarding forcing pharmaceutical companies to fund the collection and destruction of household medical waste [26–28]	Drug collection events, secured drop boxes, collection by law enforcement agencies and pharmacies [23–25, 43]	Usually DEA, law enforcement agencies and NGOs [20]. In specific counties, the Pharmaceutical companies [27]	0.01 kg/capita [44]
Canada	Most of the provinces and territories run such programs (See Table 3)	Household medical waste laws in several provinces [17, 18]	Drug collection events, secured drop boxes, and pharmacies [17, 18]	Government/pharmaceutical industry/pharmacies [17, 18]	Varies between provinces (on average 0.01-0.02 kg/capita) [18, 45, 46]
Hungary	Recyclomed [15, 47]	The law obliges the pharmaceutical industry to establish and operate a disposal system or give this duty to another organization [15]	Pharmacies and other selling points, containers in specific collection points [15]	Pharmaceutical Industry Groups [15]	0.02 kg/capita [15]
UK	No program	According to the UK environmental protection act and additional regulations, all Pharmacies are obliged to accept back unwanted medicines from patients [48].	All Pharmacies and hospitals [16, 48]. In addition, there are several local initiatives for collecting medical waste from homes [49]	Local governments [15].	Not available
Australia	RUM (Return Unwanted Medicines) [31]	Each state and territory have their own regulation regarding medicine disposal (embedded in the Drugs and Poisons Acts of each individual state)	Special containers mounted in all pharmacies [31]	Funded by the government until end of June 2018; then will be reconsidered. [31]	0.03 kg/capita [31]
Israel	No program	No legislation	HMO pharmacies are obliged to receive medicines from the public.	The Israeli Ministry of Health	13.9 % of the population [40]
- In several states, pharmacies collect household drugs on a voluntary base: Italy, Ireland, Albania, Austria, Germany, Netherland, Luxemburg, Latvia, Slovenia, Spain, Poland, Portugal, Finland, The Czech republic, Sweden, and Switzerland [3].

However, despite the clear policies and accessible information, a poll conducted in 2010 by the European Environmental Agency (EEA) shows that on average 50 % of the expired or unused drugs are not returned to pharmacies, and are flushed down toilets or sinks [3] (See also Additional file 1: Table 1).

##### Funding

The collection, transfer and treatment of household medical waste in Europe are funded by (a) The governments, for example in Sweden [17, 18], or by (b) the pharmaceutical industries, following the “polluter pays” principle, for example: The Belgian program “Bounsage” is funded by the pharmaceutical industry and distributers [19], the Spanish program “SIGRF” and also the French “Cyclamed” program, are funded by the pharmaceutical industry [17–19].

#### USA

##### Policy

There is no federal legislation regarding household medical waste collection. The issue is mostly handled by the health authorities (DEA-Drug Enforcement Administration, FDA-Food and Drugs Administration).

The FDA published detailed instructions and recommendations regarding household medical waste: the main recommendation is transferring unused/expired drugs to a DEA-authorized collector. In case no such collector is available:

1. One should mix medications (without crushing tablets or capsules) with an unpalatable substance such as dirt, kitty litter, or used coffee grounds, place the mixture in a container such as a sealed plastic bag and throw it to the garbage.
2. In case of especially harmful medication (which is listed in the FDA), the medication should be flushed down the sink or toilet as soon as they are no longer needed, in order to prevent poisoning due to uncontrolled use of the medication [20].

However, in January 2016, more than 100 environmental and health organizations, agencies, activists and state legislators (Colorado State Legislator, the California Association of Sanitation Agencies, California Product Stewardship Council, Association of Metropolitan Water Agencies, Central Marin Sanitation Agency, Environmental Services of city of West Sacramento, Minnesota Pollution Control Agency, San Francisco Department of the Environment and others) and citizens signed on a letter calling on the FDA to change its recommendation that certain medications be disposed by flushing, and to clarify that secure medication take-back programs provide the best disposal method for leftover household medications [21].

Until 2010, household medical waste was collected only by law enforcement agencies (police, Sheriff). In 2010, a rule promoted by the DEA enabled hospitals, clinics, pharmacies and drug manufactures to collect unused drugs and transfer them to medical waste treatment centers [22].

##### Medication collection systems

Today, unused drugs in the USA can be collected in several ways: Drug collection events held in accordance with the DEA [23], via secured drop boxes [24], by the law enforcement agencies [25] or by pharmacies [20] (See Table 2 for examples).

**Table 2:** Collection and recycle of unused drugs in the USA

How are unused drugs collected?	Examples
Drug collection events every several months in accordance with the DEA. The collection is free and anonymous [23].	National drug collection event in September 2015 in different US states [23], Drug collection events in Manitowoc county in Wisconsin state [25], Drug collection events in Illinois [43].
Drug collection on a monthly base	Pharmacies collect non-controlled prescription drugs and OTC medications on the last Saturday of every month in New-York state [24].
Secured drop boxes for drug collection that can be accessed in specific days and times.	Secure drop boxes in New-York state that can be accessed in specific days and times, several such drop boxes are available for the public 24 h a day, seven days a week [24].
Collection by the law enforcement agencies (Sheriff office, police)	In Manitowoc (Wisconsin) there are drug collection drop boxes in police stations [25].
Collection of unused or expired drugs by pharmacies.	Most of the states.

##### Funding

The collection, transfer and treatment of household medical waste, is usually funded by the DEA and law enforcement agencies [20]. In some cases, additional organizations fund or participate in the funding:

- In 2016 in Massachusetts, “An Act relative to substance use, treatment, education and prevention” was passed. The law follows the “polluter pays” principle, in which pharmaceutical companies fund the collection, transfer and treatment of household unused and expired drugs [26].
- Nine counties in the USA passed legislation that follows the “polluter pays” principle: seven counties in California state (Alameda, San Francisco, Santa Cruz, San Mateo, Santa Clara, Santa Barbara, and Marin) and two counties in Washington state: King county and Snohomish county [27, 28].
- In New-York state the police and the Sheriff office usually fund the drug collection and transfer. In addition, in several counties in this state, it is funded by additional organizations or county departments [24]:
  - o BRiDGES Madison Co. Council on Alcoholism and Substance Abuse (Madison county)
  - o Monroe County Department of Environmental Services
  - o Tompkins County Department of Probation
  - o Alliance for Safe Kids and Department of Environmental Facilities (Westchester county)
  - o Guthrie Environmental Health (Jefferson county)
  - o Department of Public Safety (Suffolk county)
- In Manitowoc county (Wisconsin), the drug collection programs are sponsored by a variety of agencies related to health and the environment.

#### Canada

In Canada there is variation in the policies of provinces and territories regarding the collection, transfer and treatment of unused and expired household drugs. In several provinces/territories, the pharmaceutical industry sponsors these costs according to the “polluter pays” principle [17, 18] (see Tables 1 and 3).

**Table 3:** Programs, legislations, policies, and funds regarding collection of unused and expired household drugs in Canada [17, 18]

Province/territory	Program and year of initiation	Voluntary of regulated framework	Return location	Funding sources
Alberta	ENVIRx (1988)	Voluntary	Pharmacies	Pharmaceutical companies, provincial government
British Columbia	BC MRP (1999)	Regulated under environmental management act	Pharmacies	Pharmaceutical and consumer health product industries
Manitoba	Manitoba MRP (2011)	Regulated under The Waste Reduction and Prevention Act)	Pharmacies	Pharmaceutical and consumer health product industries
New Brunswick	None		Some of the pharmacies	
Newfound-land and Labrador	Household Hazardous Waste (1998)	Voluntary	Household hazardous waste depots	Provincial and municipal governments
Nova Scotia	Medication Disposal Program (mid 1990s)	Voluntary	Pharmacies	Pharmaceutical companies
Yukon/Quebec	None		Collection events, most of the pharmacies	
Nunavut	None		Pharmacies and health centers	
Ontario	Ontario MRP (2013)	Regulated under Environmental Protection Act	Collection events, pharmacies	Pharmaceutical and consumer health product industries, Provincial government
Prince Edward Island	Take it back (2004)	Voluntary	Pharmacies and Waste Watch Drop-off centers	Island Waste Management Corporation
Saskatchewan	Pharmaceutical Waste Disposal (1997)	Voluntary	Pharmacies	Pharmacies

#### Selected central and south America countries

##### Mexico

There is no legislation regarding collection and treatment of household medical waste in Mexico [29]. However, 23 out of the 31 states in Mexico (for example, Nuevo Leon state), run the program “SINGREM” for collection and treatment of household medical waste. The program is administered by Mexico’s National Chamber of the Pharmaceutical Industry (CANIFARMA) [19, 30]. The program is sponsored by the pharmaceutical industry (companies such as Merck, Abbott and also the Israeli company TEVA) [30].

##### Brazil

There is no legislation regarding collection and destruction of household medical waste in Brazil. However, the program “Descarte Consciente”, administered by Brazil Health Service, handles the collection of household medical waste. Expired/unused medications are collected at pharmacies and the collection is not mandatory. The program is funded by the pharmaceutical industry [19].

##### Colombia

Colombia’s National Association of Entrepreneurs (ANDI) administers the program “Punto Azul” than began collecting medications in 2010 with funding supplied by pharmaceutical manufacturers and importers. Collection is located at pharmacies and large supermarkets but is not mandatory [19].

#### Australia

There is a national program in Australia for household medical collection (“Return unwanted medicine” – The “RUM” project). The program enables the public to return unwanted medications to the local pharmacies. The program is funded by the government and several pharmaceutical manufacturers [18, 31]. For additional details see Table 1.

#### Selected Middle East Countries

##### Egypt

Although there is no clear legislation regarding collection of household medical waste by pharmacies, there is evidence that pharmacies collect medical waste [32, 33]. In addition, there are Egyptian NGOs who collect unused medications and distribute them to low-income families, a model that is planned to be spread to other Arab countries [34].

##### Lebanon

There is no law in Lebanon regarding collection of household medical wasted and no organized pharmaceutical waste collection and disposal schemes in Lebanon. A survey conducted in the Beirut area showed that less than 5 % of the public return unwanted medications to pharmacies [35].

##### Saudi Arabia

In Saudi Arabia governmental healthcare facilities provide all Saudi citizens with free medication, but the awareness of individuals regarding safe drug disposal is low. Less than 5 % of the public return unwanted medications to the pharmacy or to their physicians while most of the Saudi Arabian public throw unwanted medications to the household trash, regardless of medication type [36].

### Approaches for drug collection and disposal in Israel

In Israel there is no legislation regarding the collection, transfer and treatment of household medical waste and of unused/unwanted medications (see also Table 1). However, the Israeli Ministry of Health is aware of the subject and initiated several actions, such as updating the procedures for pharmacies. In the last years there have been several steps in this field:

- In 2001 the Ministry of Health published a circular that instructed the HMO pharmacies to receive unused medications, by any person (not necessarily HMO member), free of charge. This document requires the HMOs to transfer the collected medications to pharmaceutical waste centers [37]. Accordingly, the HMOs have mounted secured iron drop-boxes for medication collection [38].
- In 2006, one leading private pharmacy in Israel initiated, in coordination with the Israeli Ministry of Health, a campaign for collecting unused medications from the public. Those who returned unused medications to the pharmacy, got in return a new pack of vitamin C.
- In 2003 the Israeli organization “Friends for health” ( “Haverim Le’refua”) was established in order to help needy populations of sick and handicapped people. One of its major projects deals with medication recycling. The organization collects unused medications from patients and their families, and transfers them to sick people. This procedure was formally added to the pharmacist ordinance, as part of a legislative amendment of the Pharmacists Ordinance. Additionally, the organization runs a pharmacy (supervised by the Ministry of Health) which receives pharmaceuticals from the pharmaceutical companies and gives them to patients [39].

In Israel, there are poor rates of medication return to the HMO pharmacies: In 2014–2015 the Central Bureau of Statistics conducted a poll which interviewed over 7000 participants (over 20 years old). The poll included also a question regarding the return rates of medications. The poll revealed that less than 14 % of the Israelis return unused medications to pharmacies [40] (Table 4).

**Table 4:** Return rates of medications to pharmacies or HMOs in 2014 [40]

City	Medication return (%)
Jerusalem	10.7
Haifa	18.6
Petach-Tikva	17.7
Ashdod	15.3
Tel-Aviv Jaffa	14.8
Rishon Letzion	12.1
Total	13.9

### Discussion – How can the current situation in Israel be improved?

In comparison to most developed countries, Israel is far behind, regarding the collection and treatment of household medical waste. In recent years there have been some improvements in this field, but the current situation in which more than 85 % of the public accumulate medications and later throw them to the garbage or flush them down the toilet or sink, endangers public health and the environment [40]. Of note, there are no data regarding the amounts and the types of medications thrown to the garbage in Israel.

A mechanism for drug collection is expensive and requires complex logistics. In addition, temporary medication storage in inappropriate facilities may cause a leak of toxic compounds on the one hand, and may be accessible to drug addicts or criminals, on the other hand. However, there is a need for a comprehensive program for safe disposal of unused medications in Israel.

There are several fields of action to cope with this issue in Israel. In the short run – raising the public awareness, and in the medium and long run – legislation and regulation in addition to extending the expiry of medications in order to reduce the amounts of thrown medications.

1. Awareness

It seems that one of the major problems in this field in Israel is the public’s lack of awareness regarding the health and environmental implications of medication accumulation and disposal. It is therefore important to raise the public’s awareness to this issue. Awareness raising tools, including publishing relevant data in the Ministry of Health website or in social networks, publicity projects, campaigns, and brochures, may be relatively easy and fast to perform within the Ministry of health without the need to collaborate or coordinate with other external entities. It is important to raise physicians’ awareness to this issue, and emphasize the importance of avoiding prescription of unnecessary medications or excessive amounts of medications. In addition, it is important that pharmacists supply detailed information regarding disposal of unused/expired medications.

2. Legislation and regulation

There is no legislation and almost no regulation regarding the collection and the treatment of household medical waste. There is a need for laws and regulations for handling this issue in Israel regarding several aspects: (a) laws that will enable collection of household medical waste by institutes in addition to HMOs: collection by pharmacies, collection drop boxes in the police, at hospitals, in the mail office and even in big supermarkets. Additionally, enacting laws to allow organization to hold drug collection events, similar to the DEA collection events held in the US, or collection of household medical waste from people's homes. (b) Individuals who pass away in retirement homes leave medications that are usually thrown to the garbage. It is important to establish a mechanism to collect and transfer the non-expired medications to needy populations [41] and the expired ones to destruction. (c) It is worthy to examine laws for incentives for returning medications to HMO or private pharmacies, such as getting pay-back money or other rewards. (d) Similarly to Europe, it is advisable to promote regulations and procedures regarding special instructions and precautions for disposal that will appear on the outer packaging of medicinal product, in the patient information leaflet or on the medication label. (e) Laws that will regulate the funding of programs for collection and destruction of medications, preferably following the “polluters pays” principle, by which, pharmaceutical companies will pay for the collection, shipment and destruction of household medical waste.

The household medical waste problem is relevant to several governmental ministries, especially the Ministry of Health (protecting the public health), and Ministry of Environmental Protection (waste collection is under the responsibility of this ministry, and also the environmental implication of household medical waste). Cooperation between these ministries is needed in order to promote this important field.

3. Funding

It is important to examine programs to sponsor the collection of household medical waste, according to the “polluter pays” principle, as done in many countries around the world. Producers, importers, distributers and pharmacies, in addition to the government, should take a part in sponsoring the collection, transfer and treatment of household medical waste. A cost/benefit analysis should be done, considering the financial costs involved in disposing drugs, and the outer costs (environmental damage, investments in advanced purification systems etc.)

## Conclusions

Less than 14 % percent of Israelis return unused pharmaceuticals. Unused drugs are a potential risk to public health, both from risk of poisoning and from environmental contamination. There is a need for a comprehensive program for safe disposal of unused medications in Israel. This program should address additional types of household medical waste such as household mercury thermometers.

## Additional file

Additional file 1: Table S1. Number of Pharmaceutical sold and returned through European return programs in 2008. (DOCX 35 kb)

## Abbreviations

DEA: Drug enforcement administration (USA)
EEA: European environmental agency
EU: European union
FDA: Food and drugs administration (USA)
HMO: Health maintenance organization
NHS: National health service (UK)
OTC: Over the counter
RUM: Return unwanted medicine (Australia)
