# Supplementary material for: Household medical waste disposal policy in Israel
Source: Isr J Health Policy Res. 2016 Oct 10;5:48. doi: 10.1186/s13584-016-0108-1 (PMC5057219; doi:10.1186/s13584-016-0108-1)
Supplement: Additional file 1: Table S1. — Number of Pharmaceutical sold and returned through European return programs in 2008. (DOCX 35 kb) [file 13584_2016_108_MOESM1_ESM.docx]

**Supplementary material**

Supplementary Table 1: Number of Pharmaceutical sold and returned through European return programs in 2008.

| Country | Number of pharmaceutical packages sold (per capita annually) | Unused/Expired pharmaceuticals collected (g per capita annually) |
| --- | --- | --- |
| Croatia | Not indicated | 0.19 |
| Estonia | 19.46 | 3.4 |
| Slovenia | 16.91 | 4.5 |
| Lithuania | 27.12 | 10 |
| Finland | 16.64 | 11 |
| Iceland | Not indicated | 19 |
| The Netherlands | 14.34 | 30 |
| Czech Republic | 25.75 | 36 |
| Liechtenstein | Not indicated | 39 |
| Belgium | 21.83 | 46 |
| Italy | 28.87 | 54 |
| Denmark | 14.84 | 55 |
| Spain | 26.99 | 57 |
| Portugal | 25.12 | 58 |
| Germany | 18.34 | 73 |
| Styria (in Austria) | 22.71 | 99 |
| Sweden | 16.91 | 119 |
| Ireland | 22.86 | 142 |
| Luxemburg | 27.72 | 174 |
| France | 51.79 | 231 |
| Switzerland | 19.78 | 237 |

Adopted from: Vollmer G. Disposal of Pharmaceutical Waste in Households – A European Survey. In: Kummerer K. Hemple M., editors. Green and Sustainable Pharmacy. Freiburg; 2010 p. 165-178.
